# Early detection of subclinical ventricular deterioration in aortic stenosis with cardiovascular magnetic resonance and echocardiography

**DOI:** 10.1186/1532-429X-15-72

**Published:** 2013-08-28

**Authors:** Seung-Pyo Lee, Sung-Ji Park, Yong-Jin Kim, Sung-A Chang, Eun-Ah Park, Hyung-Kwan Kim, Whal Lee, Sang-Chol Lee, Seung Woo Park, Dae-Won Sohn, Yeon-Hyeon Choe

**Affiliations:** 1 Cardiovascular Center, Seoul National University Hospital, 101 Daehak-ro, Jongro-gu, Seoul 110-744, Korea; 2 Department of Internal Medicine, Seoul National University College of Medicine, 101 Daehak-ro, Jongro-gu, Seoul 110-744, Korea; 3 Department of Radiology, Seoul National University College of Medicine, 101 Daehak-ro, Jongro-gu, Seoul 110-744, Korea; 4 Cardiovascular Imaging Center, Cardiac and Vascular Center, Samsung Medical Center, Sungkyunkwan University School of Medicine, 50 Irwon-dong, Gangnam-gu, Seoul 135-710, Korea

**Keywords:** Aortic stenosis, Cardiovascular magnetic resonance, Echocardiography, Heart function, Ventricular remodeling, Magnetic resonance imaging, Myocardial function, Myocardial fibrosis

## Abstract

**Background:**

Severe aortic stenosis (AS) patients with late gadolinium enhancement (LGE) on cardiovascular magnetic resonance (CMR) or left ventricular (LV) systolic dysfunction are known to have worse outcome. We aimed to investigate whether LGE on CMR would be useful in early detection of subclinical LV structural and functional derangements in AS patients.

**Methods:**

118 patients with moderate to severe AS were prospectively enrolled. Echocardiography and CMR images were taken and the patients were divided into groups according to the presence/absence of LGE and of LV systolic dysfunction (LV ejection fraction (EF) <50%). The stiffness of LV was calculated based on Doppler and CMR measurements.

**Results:**

Patients were grouped into either group 1, no LGE and normal LVEF, group 2, LGE but normal LVEF and group 3, LGE with depressed LVEF. There was a significant trend towards increasing LV volumes, worsening of LV diastolic function (E/e’, diastolic elastance), systolic function (end-systolic elastance) and LV hypertrophy between the three groups, which coincided with worsening functional capacity (all p-value < 0.001 for trend). Also, significant differences in the above parameters were noted between group 1 and 2 (E/e’, 14.6 ± 4.3 (mean ± standard deviation) in group 1 vs. 18.2 ± 9.4 in group 2; end-systolic elastance, 3.24 ± 2.31 in group 1 vs. 2.38 ± 1.16 in group 2, all p-value < 0.05). The amount of myocardial fibrosis on CMR correlated with parameters of diastolic (diastolic elastance, Spearman’s ρ = 0.256, p-value = 0.005) and systolic function (end-systolic elastance, Spearman’s ρ = -0.359, p-value < 0.001).

**Conclusions:**

These findings demonstrate the usefulness of CMR for early detection of subclinical LV structural and functional deterioration in AS patients.

## Background

Aortic stenosis (AS) is a disease that typically provokes pressure overload to the left ventricle, which left uncured, may lead to left ventricular (LV) hypertrophy, pump failure and also, to sudden cardiac death [1,2]. Surgery before the development LV dysfunction is of paramount importance for these patients. Currently, evaluation of symptomatic status and LV ejection fraction (EF) is recommended for determining surgical timing [3]. However, sudden cardiac death may occur even in patients without symptom and LV dysfunction. Furthermore, LV diastolic dysfunction [4] and exercise intolerance [5] often persist even after corrective surgery suggesting the presence of irreversible myocardial damage before surgery. These disappointing results are partly due to poor sensitivity of LVEF as a marker of myocardial damage and thus some investigators support early surgery in asymptomatic patients with normal LVEF [6]. Accordingly, early detection of myocardial damage may be clinically informative for these patients.

Before the development of overt pump failure, fibrosis of the LV myocardium ensues [7-9], which leads to diastolic function impairment [10,11] and possibly occult systolic dysfunction [12-14]. Indeed, patients with severe stenosis of the valve show significant deterioration of the diastolic properties that improves after correction of this stenosis [10,15]. More importantly, the aggravation of diastolic dysfunction is closely related to clinical outcome [16].

Late gadolinium enhancement (LGE) cardiovascular magnetic resonance (CMR) is the most accurate way to visualize the smallest focal fibrosis/scar in the myocardium [14,17]. Patients with severe AS who had LGE on CMR were more likely to experience worse outcome than those who did not [5,18-20]. Also, the degree of LGE has also been shown to correlate well with the degree of histological fibrosis in these patients [5]. In this report, we investigated whether CMR can be used to detect subclinical deterioration of the ventricular function. Especially, we focused on whether LGE-CMR would discriminate the subtle difference of cardiac function in patients with normal LVEF.

## Methods

### Patient population

A total of 118 patients with moderate to severe AS, i.e. maximal transaortic velocity >3 m/sec or mean transaortic pressure gradient >30 mmHg and aortic valve area ≤1.5cm^2^were enrolled to this prospective study from September 2009 to September 2012 at both Seoul National University Hospital and Samsung Medical Center, which was composed of a series of echocardiography and CMR. Patients were consecutively enrolled at the time of echocardiographic examination. Patients with significant concomitant valvular disease of more than mild degree, i.e. moderate aortic regurgitation or moderate mitral valve disease or a previous history of cardiac surgery or myocardial infarction were excluded. All except 9 patients underwent conventional coronary angiography or computed tomography coronary angiography for determination of significant concomitant coronary artery disease. All patients gave informed consent to the study, the protocol of which was approved by the Institutional Review Board of both institutions. Baseline laboratory tests, anthropometric measures and medical history were taken at the time of echocardiography. Body surface area was calculated with the Mosteller formula.

### Echocardiography

All patients underwent a comprehensive echocardiography with a commercial equipment (Vivid 7, GE Medical System, Horten, Norway) according to the current recommendations [21].

In brief, LV dimensions both at end-diastole and systole were measured at the standard parasternal short-axis view of papillary muscle level or parasternal long-axis view. The dimensions of the aortic root, i.e. aortic annulus, sinotubular junction and ascending thoracic aorta diameter were measured at the standard parasternal long-axis view.

Peak early and late diastolic velocity at the mitral valve tip level (E, A velocity, respectively) and mitral annular velocity (e’, a’ velocity, respectively) at the septal annulus were measured at the standard apical four-chamber view. Transaortic mean pressure gradient and maximal velocity was measured at all views possible, i.e. apical 5 or 3 chamber, subcostal, right parasternal and suprasternal notch view. Aortic valve area (AVA) was calculated using the continuity equation after acquiring time-velocity integral at the aortic valve level and also, left ventricular outflow tract level. All echocardiography measurements were averaged for three beats for patients in sinus rhythm and five beats in atrial fibrillation with baseline heart rate of <100BPM.

### Calculation of LV load and stiffness parameters

The diastolic properties of the LV were estimated using the following parameters. LV filling pressure was assessed by using a well-known surrogate parameter E/e’ [22] and to estimate the diastolic elastance of the LV (Ed), E/e’ was again divided by the stroke volume measured by CMR [23].

To estimate the systolic stiffness of LV, single beat-derived LV end-systolic elastance (Ees) was calculated as end-systolic pressure/end-systolic volume (measured by CMR) [24], where end-systolic pressure was calculated as 0.9 × (systolic blood pressure).

### Cardiovascular magnetic resonance

Cardiac magnetic resonance imaging was performed using a 3.0-T scanner with phased-array receiver coils (Sonata Magnetom, Siemens, Erlangen, Germany) under the standard protocols. In brief, steady-state free precession cine images under an adequate breath-hold were performed to visualize the LV wall motion and also, to quantify the LV function and mass. The entire LV short-axis images were acquired at a 6 mm interval from the base to apex to include the whole LV volume. The LGE images were acquired 10 minutes after intravenous gadolinium injection (0.1 mmol/kg Magnevist; Schering, Berlin, Germany). The protocol for the LGE images were as follows; slice thickness 8 mm, interslice gap 2 mm, TR 9.1 msec, TE 42 msec, flip angle 13 degrees, in-plane resolution 1.4 × 1.9 mm. Inversion delay time varied from 280 ~ 360 msec according to the time to null the normal myocardium.

LGE-CMR images were analyzed by an experienced radiologist blinded to the patients’ information. In addition, the region of myocardial fibrosis was defined as the sum of pixels with signal intensity above 5SD of the normal remote myocardium at each short-axis slice [18], using an appropriate post-processing program (CMR42, Circle Cardiovascular Imaging Inc., Calgary, Canada). Summation of the measured areas of LGE and the whole myocardium in all short-axis slices yielded the total volume of LGE and the total volume of LV. The percentage of myocardial fibrosis per total myocardium (%LGE-positive myocardium) was analyzed as the total pixels of fibrosis per total pixels of myocardium.

In addition, the pattern of LGE was classified according to the following classification. The subendocardial LGE pattern was designated if the LGE was located at the subendocardium. All other forms of LGE were located along the midwall except for one patient with linear enhancement at the epicardium. If the LGE spanned at least half of a single myocardial segment, it was defined as a linear pattern LGE or otherwise it was defined as a spot pattern LGE. The LGE pattern showing a diffuse fuzzy pattern of enhancement was defined as a patchy pattern LGE. Patients with a mixed pattern of LGE categorized above was classified according to the predominant pattern of LGE.

### Statistical analysis

Continuous variables are presented as mean (SD). The difference between two groups was compared using Student’s t-test. The difference between three groups was calculated using analysis of variance (ANOVA). Bivariate correlation analysis between the parameters of myocardial function, i.e. diastolic and end-systolic elastance, and the percentage of myocardial fibrosis per total myocardium was drawn. The results of the strength of correlation were presented as Spearman’s rho correlation coefficients because of the non-normal distribution of the percentage of myocardial fibrosis per total myocardium. Dichotomous variables are presented as number (percentage) and compared using χ2-test. All analysis was done with SPSS version 16.0 (SPSS Inc., Chicago, IL) and two-tailed p-value of <0.05 was considered statistically significant.

## Results

**Figure 1:**
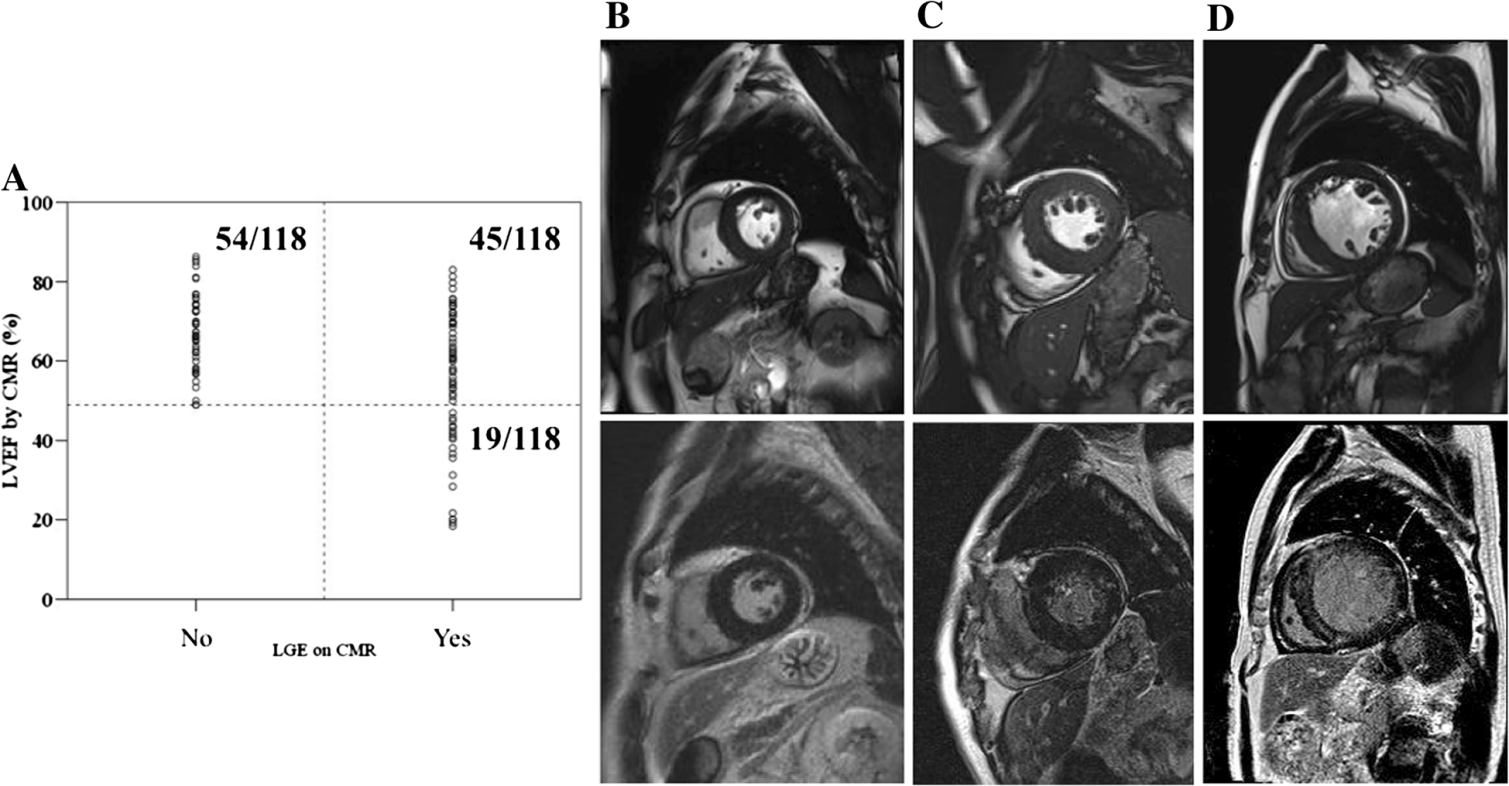
**Grouping of the study population and representative LGE-CMR images of the patients. (A)** All patients were grouped according to the presence/absence of late gadolinium enhancement (LGE) and left ventricular (LV) systolic dysfunction, defined as LV ejection fraction (EF) < 50%. Group 1 was defined as normal LVEF and no LGE on CMR (left upper quadrant), group 2 as normal LVEF with LGE on CMR (right upper quadrant), group 3 as depressed LVEF with LGE on CMR (right lower quadrant). **(B)** Representative cine and LGE-CMR images of a patient in group 1. **(C)** Representative cine and LGE-CMR images of a patient in group 2. **(D)** Representative cine and LGE-CMR images of a patient in group 3.

A total of 118 moderate to severe AS patients were prospectively enrolled for the current study (Table 1). Approximately half of these patients showed LGE on CMR and 15% of the patients had LV systolic dysfunction defined as LVEF < 50% on CMR. However, there was no patient with LV systolic dysfunction but without LGE. Therefore, the patients were grouped into three as the following; group 1, no LGE on CMR and normal LV systolic function, group 2, LGE on CMR but normal LV systolic function, and group 3, LGE on CMR and also depressed LV systolic function (Figure 1).

**Table 1:** Baseline clinical characteristics of the study participants

	**Total (n = 118)**	**Group 1 (n = 54)**	**Group 2 (n = 45)**	**Group 3 (n = 19)**	**p-value**
Age (years)	68 (10)	68 (10)	68 (10)	69 (11)	0.929
Male, n (%)	34 (50.0)	23 (56.1)	11 (40.7)	11 (40.7)	0.215
Systolic blood pressure (mmHg)	125 (18)	128 (18)	125 (16)	118 (20)	0.108
Diastolic blood pressure (mmHg)	69 (11)	70 (12)	69 (12)	66 (9)	0.349
Body surface area (m^2^)	1.65 (0.17)	1.65 (0.16)	1.67 (0.18)	1.63 (0.17)	0.681
Baseline creatinine (mg/dL)	0.93 (0.23)	0.92 (0.25)	0.92 (0.20)	0.95 (0.22)	0.877
Hypertension, n (%)	68 (57.6)	33 (61.1)	25 (55.6)	10 (52.6)	0.763
Diabetes mellitus, n (%)	29 (24.6)	10 (18.5)	13 (28.9)	6 (31.6)	0.364
Hyperlipidemia, n (%)	25 (21.2)	17 (31.5)	6 (13.3)	2 (10.5)	0.410
Current smoker, n (%)	22 (18.6)	6 (11.1)	8 (17.8)	8 (42.1)	0.011
Atrial fibrillation, n (%)	10 (8.5)	5 (9.3)	2 (4.4)	3 (15.8)	0.317
Coronary artery disease, n (%)^§^	16 (14.7)	8 (16.3)	5 (12.2)	3 (15.8)	0.849

All patients were grouped according to the presence/absence of late gadolinium enhancement (LGE) and left ventricular (LV) systolic dysfunction, defined as LV ejection fraction (EF) < 50%. Group 1 was defined as normal LVEF and no LGE on CMR, group 2 as normal LVEF with LGE on CMR, group 3 as depressed LVEF with LGE on CMR. The difference of baseline clinical characteristics between the three groups was calculated using analysis of variance (ANOVA) and the results presented as p-value. NYHA, New York Heart Association. The data are presented as mean (SD) or number (percentage). ^§^The evaluation of the presence/absence of coronary artery disease was evaluated in 109 patients.

Baseline clinical characteristics are summarized in Table 1. In brief, there were no significant differences in basic anthropometric measures except for the smoking status. All except 5 patients in group 1 and 4 patients in group 2 underwent evaluation for the presence of significant concomitant coronary artery disease but there was no difference.

The baseline echocardiography parameters are summarized in Table 2. Compared with group 1 and 2, the patients in group 3 had significantly larger LV dimensions, shorter deceleration time and smaller mitral septal annular velocity. Also as a consequence of LV systolic dysfunction in group 3, the peak transaortic velocity was smaller on Doppler echocardiography. There was a tendency towards thicker interventricular septum and posterior wall thickness in group 2 compared with group 1. On CMR, there were significant differences in all parameters, including LV volumes, LV systolic function, cardiac index and also, LV mass (Table 3) between group 1, 2 and 3.

**Table 2:** Echocardiographic parameters of the study participants

	**Total (n = 118)**	**Group 1 (n = 54)**	**Group 2 (n = 45)**	**Group 3 (n = 19)**	**p-value**
LVEDD (mm)	51 (5)	49 (5)	50 (5)	59 (7)^‡^	<0.001
LVESD (mm)	33 (8)	30 (4)	31 (4)	46 (9)^‡^	<0.001
IVST (mm)	12 (5)	11 (2)	13 (6)^*^	13 (6)	0.080
PWT (mm)	11 (2)	10 (2)	11 (2)^*^	11 (2)	0.036
Aortic annulus diameter (mm)	21 (2)	21 (2)	21 (3)	22 (3)	0.902
E (m/sec)	0.77 (0.28)	0.73 (0.23)	0.79 (0.28)	0.83 (0.40)	0.386
DT (m/sec)	252 (78)	246 (71)	270 (81)	226 (85)^†^	0.088
e’ (cm/sec)	4.8 (1.6)	5.3 (1.7)	4.7 (1.3)	3.9 (1.6)^†^	0.005
Vmax (m/sec)	4.7 (0.8)	4.6 (0.8)	4.9 (0.8)^*^	4.3 (0.7)^‡^	0.015
AVA (cm^2^)	0.76 (0.21)	0.79 (0.20)	0.75 (0.21)	0.70 (0.25)	0.291
AVA index (cm^2^/m^2^)	0.46 (0.13)	0.48 (0.13)	0.45 (0.12)	0.43 (0.14)	0.234
Transaortic mean PG (mmHg)	54 (22)	52 (21)	59 (19)	50 (28)	0.152

The data are presented as mean (SD). The difference of each parameters between the three groups was calculated using analysis of variance (ANOVA) and between the two groups with Student’s t-test and the results presented as p-value. LV, left ventricle; EDD/ESD, end-diastolic/systolic diameter; IVST, interventricular septal thickness; PWT, posterior wall thickness; E, early diastolic velocity at mitral valve tip; DT, deceleration time; e’, early mitral annular velocity at the septal annulus; Vmax, maximal transaortic velocity; AVA, aortic valve area; PG, pressure gradient.^*^p < 0.01 versus group 1, ^†^p < 0.01 versus group 2,^‡^p < 0.05 versus group 2.

**Table 3:** CMR parameters of the study participants

	**Total (n = 118)**	**Group 1 (n = 54)**	**Group 2 (n = 45)**	**Group 3 (n = 19)**	**p-value**
LV end-diastolic volume index (mL/m^2^)	98.9 (33.5)	84.7 (22.9)	97.3 (25.1)^**^	143.4 (38.9)^‡^	<0.001
LV end-systolic volume index (mL/m^2^)	41.0 (30.1)	28.5 (13.2)	35.1 (16.5)^*^	90.6 (39.3)^‡^	<0.001
LV ejection fraction (%)	61.4 (15.1)	67.3 (9.8)	65.3 (9.7)	35.2 (10.6)^‡^	<0.001
LV cardiac index (L/min/m^2^)	3.94 (1.07)	3.72 (1.03)	4.35 (1.05)^**^	3.50 (0.88)^‡^	0.002
LV mass index (g/m^2^)	100.8 (37.4)	86.0 (29.6)	108.0 (40.9)^**^	125.5 (31.2)	<0.001
LGE(+) myocardium/total myocardium (%)	4.18 (5.75)	0	6.91 (5.03)	9.60 (7.16)	<0.001

The data are presented as mean (SD). The difference of each parameters between the three groups was calculated using analysis of variance (ANOVA) and between the two groups with Student’s t-test and the results presented as p-value. LV, left ventricle; LGE, late gadolinium enhancement.^*^p < 0.01 versus group 1, ^**^p < 0.05 versus group 1,^‡^p < 0.05 versus group 2.

**Figure 2:**
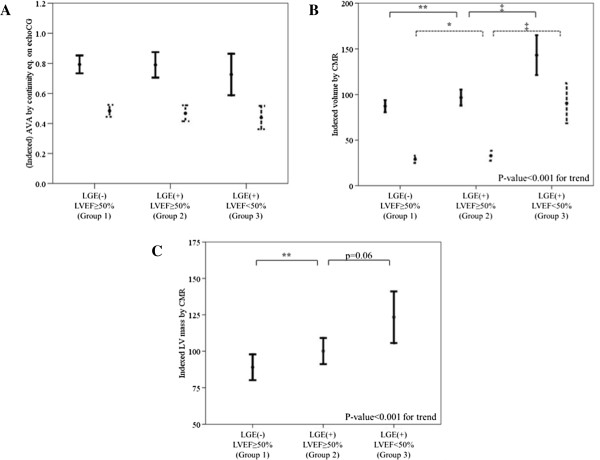
**Structural LV remodeling assessed with CMR according to the group. (A)** No significant trend in AVA (solid line) nor indexed AVA (dotted line) according to the group of patients. **(B)** Significant trend in both indexed LV end-diastolic volume (solid line) and indexed LV end-systolic volume (dotted line) according to the group of patients. P-value for trend <0.001 in both parameters. **(C)** Significant trend in indexed LV mass according to the group of patients. ^*^p < 0.01 versus group 1, ^**^p < 0.05 versus group 1, ^‡^p < 0.05 versus group 2.

Although there was no significant difference in the AVA between the three groups (Figure 2A), stepwise differences were noted between the three groups in both end-diastolic and end-systolic volumes resulting in a significant trend towards larger LV in group 3 (Figure 2B). There was also significant difference in the degree of hypertrophy between the three groups (Figure 2C). Interestingly, although there was no significant difference in the EF between group 1 and 2 (Table 3), the volumes and mass in group 1 and 2 were notably different, suggesting subclinical adverse LV remodeling in group 2 (Figure 2B and 2C).

**Figure 3:**
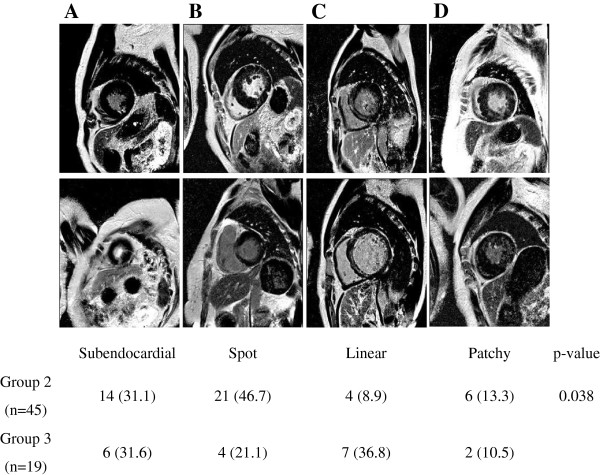
**Diverse pattern of LGE in the cohort. (A)** A subendocardial pattern of LGE. **(B)** A spot pattern LGE with single fibrosis region or multiple fibrosis areas. **(C)** A linear pattern LGE, spanning at least half of a myocardial segment. **(D)** A patchy pattern LGE. p-value = 0.038 for χ^2^-test.

The pattern of LGE was classified according to the location and the span width of the LGE (Figure 3). Although the proportion of patients with subendocardial LGE pattern (Figure 3A) was similar in group 2 and 3 (14/45 (31.1%) in group 2 vs. 6/19 (31.6%) in group 3), the proportion of patients with spot pattern (Figure 3B) was lower in group 3 than group 2 (21/45 (46.7%) in group 2 vs. 4/19 (21.1%) in group 3). On the contrary, the linear LGE pattern (Figure 3C) was more frequent in group 3 (4/45 (8.9%) in group 2 vs. 7/19 (36.8%) in group 3). The proportion of patients with patchy pattern (Figure 3D) were similar between the two groups (6/45 (13.3%) in group 2 vs. 2/19 (10.5%) in group 3). Altogether, the pattern of LGE was slightly different between the two groups (p-value = 0.038).

**Figure 4:**
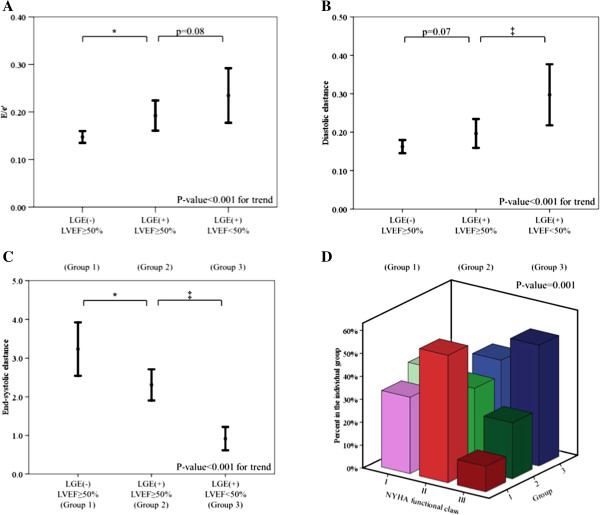
**Functional LV remodeling assessed with CMR according to the group. (A)** Significant trend in E/e’, a parameter of LV filling pressure, according to the group of patients. **(B)** Significant trend in diastolic elastance (Ed), a parameter of diastolic LV chamber stiffness, according to the group of patients. **(C)** Significant trend in end-systolic elastance, a parameter of systolic LV chamber stiffness, according to the group of patients. P-value for trend <0.001 for all parameters.^*^p < 0.01 versus group 1, ^‡^p < 0.05 versus group 2. **(D)** The functional status of each patient was graded according to the New York Heart Association (NYHA) classification. A significant trend was found in NYHA functional class according to the group of patients. See Figures 1 and 2 for other symbols or abbreviations.

Next, we analyzed whether there was any difference between the three groups in the functional remodeling parameters of the LV, i.e. diastolic and systolic function, which is summarized in Table 4. Left ventricular filling pressure, as estimated by E/e’ and diastolic elastance, Ed were compared. There was a significant trend towards worse diastolic function as shown by the gradual increase of E/e’ and Ed by the groups (Figure 4A and 4B). In terms of end-systolic elastance (Ees), there was also a significant trend towards decreasing end-systolic elastance and also, significant difference in the end-systolic elastance between the 3 groups (Figure 4C). It was also notable that the stiffness parameters, Ed and Ees, in group 1 and 2 were different, which suggested advanced LV functional impairment in group 2 (Figure 4B and 4C). Altogether, there was a significant trend towards worse functional capacity (Figure 4D).

**Table 4:** Left ventricular (LV) chamber stiffness parameters of the study participants

	**Total (n = 118)**	**Group 1 (n = 54)**	**Group 2 (n = 45)**	**Group 3 (n = 19)**	**p-value**
E/e’	17.3 (8.3)	14.6 (4.3)	18.2 (9.4)^*^	22.9 (10.8)	<0.001
Ed (mL-1)	0.20 (0.12)	0.17 (0.06)	0.20 (0.12)	0.31 (0.16)^‡^	<0.001
Ees (mmHg/mL)	2.54 (1.91)	3.24 (2.31)	2.38 (1.16)^*^	0.93 (0.56)^‡^	<0.001

The data are presented as mean (SD). The difference of each parameters between the three groups was calculated using analysis of variance (ANOVA) and between the two groups with Student’s t-test and the results presented as p-value. Ed, diastolic elastance; Ees, end-systolic elastance.^*^p < 0.01 versus group 1, ^‡^p < 0.05 versus group 2.

**Figure 5:**
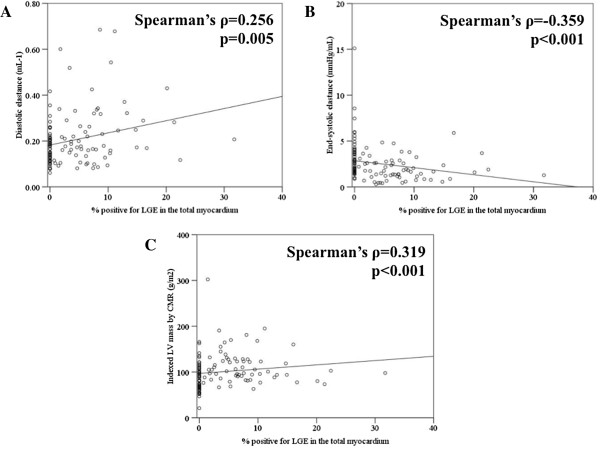
**Correlation of LV functional parameters and LV mass with the degree of myocardial fibrosis.** LGE was considered present when the signal intensity of the index myocardial segment was greater than 5SD of the remote normal myocardial signal. **(A)** Positive correlation between the degree of LGE and diastolic LV chamber stiffness (diastolic elastance, Ed), **(B)** negative correlation between the degree of LGE and systolic LV chamber stiffness (end-systolic elastance, Ees) and **(C)** positive correlation between the degree of LGE and indexed LV mass.

To dissect the association of the degree of myocardial fibrosis with LV functional remodeling, correlation between the % fibrosis and diastolic stiffness parameter, Ed or systolic stiffness parameter, Ees was drawn. There was a positive correlation between Ed and % LGE-positive myocardium (Figure 5A, Spearman’s ρ = 0.256, p = 0.005), in contrast to negative correlation between Ees and % LGE-positive myocardium (Figure 5B, Spearman’s ρ = -0.359, p < 0.001). In addition, there was a positive correlation between E/e’ and % LGE-positive myocardium (Spearman’s ρ = 0.233, p = 0.012) and between e’ and % LGE-positive myocardium (Spearman’s ρ = -0.248, p = 0.007). Also, there was a positive correlation between LV mass index by CMR and % LGE-positive myocardium (Figure 5C, Spearman’s ρ = 0.319, p < 0.001). However, all of the correlation degrees were weak. The correlation between % LGE-positive myocardium and the AS severity was non-significant (Spearman’s ρ = -0.139, p = 0.134 for indexed AVA; Spearman’s ρ = -0.042, p = 0.650 for transaortic mean pressure gradient; Spearman’s ρ = 0.089, p = 0.340 for peak transaortic velocity).

## Discussion

By dividing the study population into 3 groups according to the presence/absence of LGE and LV systolic dysfunction on CMR, parameters predictive of outcome in severe AS patients [5,18-20,25], we could show that there is a trend towards adverse structural and functional remodeling in severe AS patients if there is LGE and LV systolic dysfunction on CMR. More importantly, even if the LVEF was normal, those with LGE on CMR had significantly stiffer LV compared with those without, which suggests that LGE-CMR may be useful for early detection of subclinical LV structural and functional deterioration in severe AS patients with normal LVEF.

### Basic pathophysiologic mechanism underlying the LV response to AS

The LV loaded with chronic pressure undergoes hypertrophy according to the Laplace’s law, which is also accompanied by elevation of end-diastolic pressure [26]. Following this, the myocardial perfusion pressure falls [27] and subsequently, myocardial ischemia ensues. The eventual endpoint of this cascade is cardiomyocyte apoptosis, myocardial fibrosis replacing the apoptotic myocytes [7] and subsequently, gradual deterioration of the LV systolic function. In our cohort of patients, none of the patients had LV systolic dysfunction but no LGE. In other words, patients with LV systolic dysfunction had some degree of LGE in the myocardium. These findings support a general cascade of ‘Aortic valve stenosis → LV hypertrophy → Myocardial fibrosis → Ventricular stiffness’.

Previous studies in AS patients have demonstrated that the degree of myocyte apoptosis and myocardial fibrosis correlates well with the degree of LV systolic function deterioration [7]. Also, the degree of myocardial fibrosis detected by endomyocardial biopsy tended to show a significant trend to myocyte hypertrophy and LV dilatation [5]. These findings verify that the above concepts are indeed the situation that occurs in the setting of AS.

Analysis of our cohort demonstrated a significant trend towards increase of LV volume, mass and LV systolic/diastolic stiffness according to the presence of LGE and LVEF depression, which is in line with the above concepts and findings. This trend in our paper provides a proof-of-concept about the structural pathophysiologic response in AS and how myocardial fibrosis is related to the LV remodeling process.

### Myocardial function in severe AS and its relationship with LGE

Several previous papers have demonstrated that LVEF may not be an optimal parameter for reflecting the true myocardial function in severe AS [28,29]. More specifically, there are a substantial proportion of patients with normal EF that display significant depression of myocardial contractility [12-14] and subsequently a worse prognosis [28,30]. In this aspect, our paper provides direct evidence that even focal myocardial scar demonstrated on CMR may be related to ventricular stiffness in both diastolic and systolic phase, which is line with a previous paper demonstrating a similar finding [5]. As discussed above, the demonstration of focal scar may be a marker of the degree of cardiomyocyte apoptosis, which in turn may turn out to be deterioration of the systolic and diastolic ventricular function.

It has been suggested that the midwall mechanics of LV may be the determinant of myocardial contractility in AS [31]. Although the patterns of LGE are diverse in patients with AS, it was interesting to see a significant proportion of patients with midwall fibrosis [20] in our cohort. Moreover, patients with overt LV systolic dysfunction (LVEF < 50%) were more likely to demonstrate a linear pattern of LGE in the midwall, suggesting an important role of midwall contractility. Importantly, a recent paper has nicely shown that the midwall LGE carries a similar risk of mortality as the infarct pattern LGE [20], which could be explained by the depression of the myocardial contractility.

### Evidence of diverse LV response in severe AS

Although the general concept of ‘Aortic valve stenosis → LV hypertrophy → Myocardial fibrosis → Ventricular stiffness’ has been previously reviewed in previous papers [26] and our analysis as well, the results of our paper also demonstrated that the correlation between each step may not be robust. For example, there was no significant correlation between the degree of aortic valve area and myocardial fibrosis, which suggest that aortic stenosis *per se* may be only one of the contributing factors accelerating myocardial fibrosis.

Although only a few papers have addressed this question directly, factors such as age [5], diabetes [11] and genetic polymorphism [32] have all been suggested as ‘contributors’ of myocardial fibrosis. The pace of AVA change may also affect the degree of LVH. Furthermore, previous study has also demonstrated that the correlation between AVA and the degree of LVH may be poor [33]. These suggest that the process directing ‘Aortic valve stenosis → Myocardial fibrosis’ is a multifactorial process involving various factors in between and that the severity of AS should also take into account the ventricular response to pressure overload rather than the numerics associated with the valve itself [34]. Also, in this context, parameters directly assessing diffuse myocardial fibrosis such as postcontrast T1 values [35] or T1 mapping with various CMR sequences [36] and its correlation with ventricular function are awaited in the future.

### Utility of CMR in detection of subclinical myocardial dysfunction

One of the most interesting and novel finding in this paper is that even in patients with normal systolic function, patients with LGE tend to have stiffer LV chamber, elevated E/e’ and Ed and lower Ees. These findings tell us that even in patients with normal EF, a process of subclinical LV dysfunction ensues in patients with LGE on CMR. Although there have been reports demonstrating the prognostic value of LGE on CMR in patients with AS [5,18,20], the guidelines dealing with the timing of intervention uses only LVEF as the criteria for surgery. As suggested in a recent review of adjunct criteria for assessment of AS [29], our data suggests that the result of LGE-CMR may be integrated as an adjunct criteria for surgical intervention in these group of patients.

### Limitations of the study

Our paper is not without limitations. First, the size of the population was not large. However, the accurate assessment of LV volume, function and mass by CMR demonstrated that, in spite of the small sample size, there was a significant difference in these parameters between patients with versus without LGE. Moreover, our cohort is one of the largest reports so far in terms of AS assessed with CMR. Furthermore, out cohort is the largest one to combine two imaging modalities simultaneously for assessing the remodeling of LV in AS patients. Second, we cannot provide a definite clinical implication as to whether the stiffness parameters are predictors of outcome in these patients. However, there was a significant trend toward worse functional capacity in patients with LGE and LV systolic dysfunction, which suggests that patients with LGE may do worse than those without as in previous papers [5,19,20]. We do think that more data is desperately needed on the functional outcome in the future. Third, the degree of LGE was not matched with the histological findings. However, it has been persistently shown by previous papers that the degree of LGE shows robust correlation with the degree of histological fibrosis [5,18].

## Conclusion

In conclusion, our analysis results demonstrate that with the use of CMR, it may be possible to detect subclinical LV structural and functional deterioration in moderate to severe AS patients. The efficacy of studying LV remodeling comprehensively in predicting the ventricular structural and functional remodeling in these patients warrants further in-depth investigation.

## Competing interests

The authors declare that they have no competing interests.

## Authors’ contributions

SPL designed the study, gathered, analyzed, interpreted the data and wrote the manuscript. SJP and YJK designed the study, gathered, analyzed, interpreted the data and approved the final version of the manuscript. SAC, EAP, HKK, WL, SCL, SWP, DWS and YHC gathered data and revised the manuscript critically for important intellectual contents. All authors read and approved the final manuscript.
